# Respiratory Syncytial Virus Incidence in Young Children in the United States: Impact of Methodologies and Patient Characteristics

**DOI:** 10.1111/irv.70094

**Published:** 2025-04-03

**Authors:** Sabina O. Nduaguba, Phuong T. Tran, Renata Shih, Lyn Finelli, Yoonyoung Choi, Yanning Wang, Almut G. Winterstein

**Affiliations:** ^1^ Department of Pharmaceutical Outcomes and Policy, College of Pharmacy University of Florida Gainesville Florida USA; ^2^ Department of Pharmaceutical Systems and Policy School of Pharmacy Morgantown West Virginia USA; ^3^ West Virginia University Cancer Institute Morgantown West Virginia USA; ^4^ Faculty of Pharmacy HUTECH University Ho Chi Minh City Vietnam; ^5^ Congenital Heart Center University of Florida Gainesville Florida USA; ^6^ Center for Observational and Real‐World Evidence Merck & Co., Inc Rahway New Jersey USA; ^7^ Center for Drug Evaluation and Safety University of Florida Gainesville Florida USA; ^8^ Department of Health Outcomes and Biomedical Informatics, College of Medicine University of Florida Gainesville Florida USA; ^9^ Department of Epidemiology, College of Medicine and College of Public Health and Health Professions University of Florida Gainesville Florida USA

**Keywords:** epidemiologic methods, incidence, respiratory syncytial virus infection

## Abstract

**Objectives:**

This study aimed to estimate the incidence of respiratory syncytial virus (RSV) infections in US inpatient and outpatient settings.

**Methods:**

We established national cohorts of privately insured children < 5 years (2011–2019) to estimate annual and seasonal incidences of lower respiratory tract infection (LRTI), RSV‐LRTI, and RSV acute respiratory infection (RSV‐ARI), stratified by age and high‐risk conditions per American Academy of Pediatrics definitions. Sensitivity analyses varied episode definitions and assessed the impact of immunoprophylaxis and RSV under‐ascertainment.

**Results:**

Among 6,767,107 children, annual RSV‐LRTI rates dropped with increasing age in both inpatient (7.9 for age < 1 year to 0.2 for age 4 per 1000 person‐years) and outpatient settings (48.3 to 1.6). Most RSV‐ARI (~80%–90%) was RSV‐LRTI. RSV‐LRTI accounted for > half of LRTI hospitalizations among infants (7.9 RSV‐LRTI versus 14.7 LRTI) and for ~20% outpatient LRTI (48.3 versus 250.3), but this contribution declined with older age. Outpatient RSV‐LRTI was > 5 times inpatient rates.

Inpatient RSV‐LRTI rates dropped consistently with increasing gestational age (GA) (35.6 for GA < 29 weeks versus 7.6 for term infants), while outpatient rates were similar across GA groups (54.0 versus 51.6). Infants with Down syndrome had the highest RSV‐LRTI rates, and any high‐risk group had rates >2 times higher than healthy term infants. Across all strata, seasonal rates were > 2 annual rates. Modeling suggested that claims data captured 42% of all RSV episodes.

**Conclusion:**

This study provides national, population‐based estimates of medically attended RSV infections across age groups and high‐risk strata. Results allow granular assessments of disease burden to guide recommendations for new RSV prevention strategies.

## 1 Introduction

Respiratory syncytial virus (RSV) is a major cause of lower respiratory tract infection (LRTI) in young children. In 2019, RSV was estimated to cause 33.0 million LRTIs, 3.6 million hospitalizations and 101,000 deaths in children < 5 years globally [1]. Treatment of RSV infections is symptomatic and prevention strategies focus on immunoprophylaxis, currently available in the United States as a short‐acting monoclonal antibody, palivizumab and an extended half‐life monoclonal antibody. Due to high costs and moderate efficacy of prophylaxis [2, 3, 4, 5, 6, 7, 8, 9, 10, 11], palivizumab is typically recommended only for certain high‐risk groups [12, 13, 14, 15, 16, 17]. No policy recommendations have yet been made for nirsevimab.

While various clinical and environmental risk factors for RSV‐LRTI have been identified, infections in healthy infants contribute most of the disease burden. Most published incidence data come from studies of children ≤ 2 years of age and have investigated RSV‐LRTIs requiring hospitalization; there is less data describing the epidemiology of RSV‐infection in the broader population of young children, including milder infections attended in ambulatory care [2, 3, 4, 7, 8, 9, 10].

Previous studies have used different methodologic approaches to quantify disease burden, reporting either seasonal or annual incidence estimates, defining varying criteria to delineate unique infection episodes, or ignoring the impact of immunoprophylaxis in decreasing infection risk. Commonly, studies reporting incidence rates do not measure the time that each patient contributes to the observation window, which is particularly important when infants are born during the RSV season. Direct contrast between these diverse approaches in a population‐based sample of children would be valuable to highlight their relative impact on incidence estimates. A comprehensive description of overall disease burden of RSV will also assist in evaluating the public health impact of emerging agents for immunoprophylaxis.

We estimated seasonal and annual incidence rates of community‐acquired LRTI, RSV‐associated LRTI (RSV‐LRTI), and RSV‐associated acute respiratory infection (RSV‐ARI) in inpatient or outpatient settings. We evaluated the impact of different approaches to delineate unique infection episodes and accounted for palivizumab exposure, stratified by year, geographic region, chronological age, gestational age (GA), and key high‐risk conditions discussed in the current American Academy of Pediatrics (AAP) guidelines [12].

## 2 Methods

### 2.1 Data Sources

The study population was established from enrollees in the MarketScan® Commercial Claims and Encounters Database 2011–2019. MarketScan includes individuals with employer‐sponsored health insurance across US geographic regions. It allows longitudinal follow‐up with detailed information about medical encounters and outpatient prescription drug dispensing. The University of Florida Institutional Review Board exempted this study from review because of the use of de‐identified data.

### 2.2 Study Design

We estimated incidence rates of RSV infections among children < 5 years. For annual estimates, children entered the cohort on July 1st, when born or at enrollment in a non‐capitated health and drug plan captured in MarketScan, when they were in ambulatory care (e.g., for newborns after hospital discharge), and for risk group‐specific estimates, when they met inclusion criteria specific to a high‐risk stratum (e.g., birthday for age‐specific estimates), whichever occurred last. Cohorts were followed until June 30th in the following year, health plan dis‐enrollment, hospitalization for non‐outcome causes, or, for high‐risk condition strata, at the end of the high‐risk condition period, whichever occurred first. Both non‐RSV and RSV‐related hospitalized children were allowed to reenter the cohort once discharged if all other eligibility criteria were met. For season‐specific incidence estimates, we constrained follow‐up to a core RSV season from November to February. We chose a core season for simplicity, that is, to avoid the need to determine geography‐ and year‐specific season onset and offset (see Supplementary Material and Supplementary Excel File S1).

Three outcomes were evaluated, including LRTI, LRTI with RSV diagnosis (RSV‐LRTI), and any acute (upper and lower) respiratory infection with RSV diagnosis (RSV‐ARI). These outcomes were classified according to the relevant healthcare setting in which care was delivered as inpatient, intensive care unit (ICU), outpatient, and emergency department. They were measured using International Classification for Disease, Clinical Modification (ICD‐CM) diagnosis codes in any position for outpatient and emergency department visits. To ensure the infection was the reason for hospital admission, we required a principal diagnosis code of LRTI or ARI, or a secondary diagnosis of LRTI or ARI with a primary diagnosis of a medical condition that is directly related to the infection, such as respiratory distress (Supplemental Excel File S1).

#### 2.2.1 Delineation of Unique Infection Episodes

**FIGURE 1:**
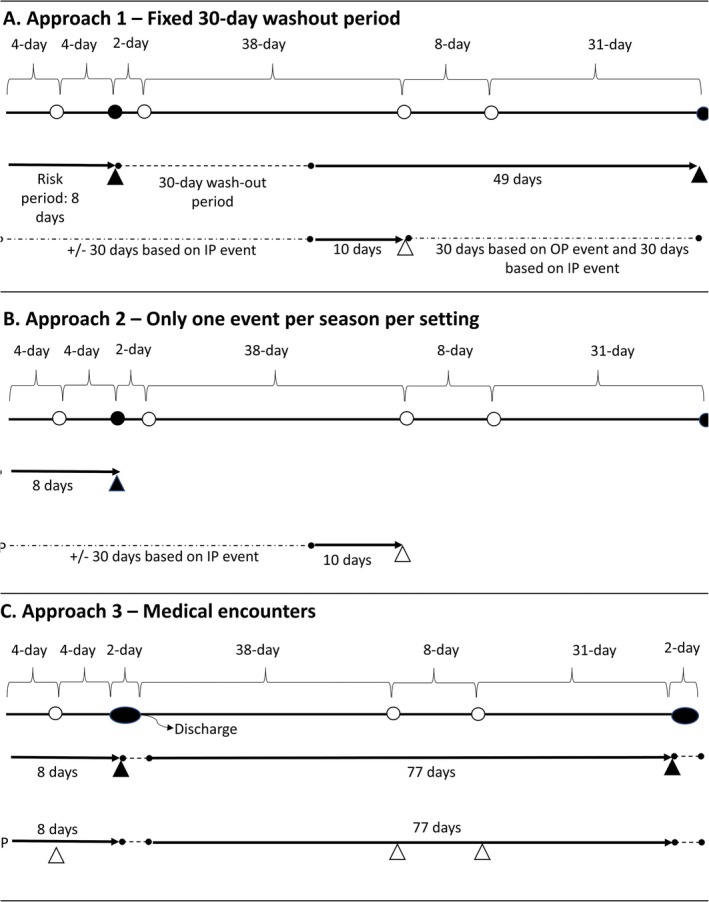
Illustration of identification of distinct episodes among RSV‐specific encounters. A round shape (⚫ or ⚪) is a medical encounter; a triangle shape (▲ or △) is a counted encounter/episode. An ellipse shape (⬬) represents hospitalization with a length of stay. Black is inpatient; white is outpatient. A dotted line is the washout period. An arrow is an at‐risk period. Abbreviations: IP, inpatient; OP, outpatient.

To capture distinct RSV episodes, we considered encounters that occurred a minimum of 30 days apart and applied a hierarchical approach where hospitalizations were prioritized over outpatient episodes, each layer applying a washout period of 30 days (Approach 1; Figure 1). To explore potential misclassification of unique episodes, we tested two additional approaches where only the first encounter in each year/season was counted, again prioritizing inpatient over outpatient episodes (Approach 2), and where every encounter was counted (Approach 3).

#### 2.2.2 Stratifications

For each outcome and each approach, we stratified incidence estimates by year, chronological age, gestational age, presence of chronic lung disease (CLD), congenital heart disease (CHD), anatomic pulmonary abnormalities or neuromuscular disease, Down syndrome, cystic fibrosis, and profound immunosuppression. Definitions of high‐risk conditions employed clinical and age criteria from the most recent AAP guidelines [12] (Supplemental Material and Supplemental Excel File S1) [18, 19, 20]. For analyses of GA‐specific subgroups, we restricted the sample to enrollees in October 2015 to December 2019 to capitalize on superior capture of gestational age in the ICD‐CM Version 10 [21]—introduced in October 2015.

#### 2.2.3 Immunoprophylaxis

We determined palivizumab exposure using outpatient encounter claims indicating palivizumab administration or a combination of a palivizumab pharmacy claims and the closest outpatient encounter within ± 10 days. Exposure started at the date of medical encounter and was assumed to last for 30 days (Supplemental Excel File S1).

## 3 Statistical Analyses

Crude annual incidence rates were expressed as the number of qualifying events per 1000 person‐years of follow‐up [22]. For ease of comparison to some previously published work, we also report incidence estimates with the number of patients as the denominator (incidence proportion), considering each child who contributed ≥ 1 day to the respective risk period.

To account for the protective effect of RSV immunoprophylaxis, we calculated adjusted RSV‐LRTI incidence rates for infants with high‐risk conditions, and for all children < 2 years stratified by gestational age. Because palivizumab administration is not ascertainable from claims data during hospitalization, we conservatively considered the first 30‐day period after a hospital discharge a palivizumab nonexposed period and conducted a sensitivity analysis, where we assumed all high‐risk children received prophylaxis at discharge. Rates attributed to the palivizumab‐exposed periods were upward‐adjusted based on efficacy estimates for preterm infants (78% reduction in RSV‐LRTI hospitalization, i.e., adjusted rate = observed rate/[1–0.78]), infants with CHD (45%), and CLD (39%) [23, 24]. We assumed a 50% reduction of RSV‐hospitalization for RSV episodes attended in outpatient care and the remaining high‐risk conditions for which efficacy data were not available [25].

Finally, to account for RSV under‐ascertainment, we developed a model using National Respiratory and Enteric Virus Surveillance System (NREVSS) data from the US Centers for Disease Control and Prevention to predict the number of LRTI events attributable to RSV [26]. In brief, the prediction model uses weekly reported RSV and influenza positivity rates collected in NREVSS to fit regression curves on observed all‐cause LRTI rates. The contribution of RSV to LRTI rates is then calculated as the difference between the predicted LRTI rates with RSV positivity results and with RSV positivity set to zero [26]. Because NREVSS does not provide test positivity results specific to age groups or settings, this adjustment was only conducted for a pooled incidence estimate considering unique inpatient and outpatient episodes across all study age groups. All analyses were conducted using SAS 9.04.01.M6.

## 4 Results

A total of 6,767,107 children and 9,831,577 person‐years were available for analysis. Across all three outcomes and medical settings, crude incidence rates dropped consistently with increasing chronological age (Tables 1 and 2 and interactive eFigure1 [see https://bit.ly/41bpz4u]). For example, the annual incidence of LRTI, RSV‐LRTI, and RSV‐ARI, episodes requiring hospitalization decreased from age < 1 to 4 from 14.7 to 1.9, 7.9 to 0.16, and 9.3 to 0.3 per 1000 person‐years, respectively. Similarly, in the outpatient setting, incidence rates decreased with age from 250.3 to 110.3, 48.2 to 1.6, and 57.9 to 2.0 per 1000 person‐years for LRTI, RSV‐LRTI, and RSV‐ARI respectively.

**TABLE 1:** Comparison of annual inpatient LRTI, RSV‐ARI, and RSV‐LRTI incidence estimates per 1000 person‐years by chronological age, and approach to delineate unique episodes.

	Approach 1 (30‐day washout)			Approach 2 (1 episode/year)			Approach 3 (encounter‐based)		
Age	Incidence (total IP) (95% CI)	Incidence (ICU) (95% CI)	Risk years	Incidence (total IP) (95% CI)	Incidence (ICU) (95% CI)	Risk years	Incidence (total IP) (95% CI)	Incidence (ICU) (95% CI)	Risk years
LRTI									
< 1 year	14.69 (14.51–14.86)	3.21 (3.13–3.29)	1,832,494	14.24 (14.07–14.41)	3.08 (3.01–3.17)	1,821,842	15.40 (15.23–15.58)	3.32 (3.24–3.41)	1,836,507
1–< 2 years	7.39 (7.27–7.51)	1.41 (1.35–1.46)	1,892,566	7.05 (6.93–7.17)	1.31 (1.26–1.36)	1,887,496	7.85 (7.72–7.97)	1.39 (1.34–1.44)	1,894,320
2–< 3 years	3.76 (3.68–3.85)	0.27 (0.24–0.29)	1,964,522	3.64 (3.55–3.72)	0.25 (0.23–0.27)	1,961,876	3.97 (3.88–4.06)	0.25 (0.23–0.27)	1,965,712
3–< 4 years	2.61 (2.54–2.68)	0.17 (0.15–0.19)	2,031,088	2.50 (2.43–2.57)	0.16 (0.14–0.17)	2,029,263	2.70 (2.63–2.77)	0.15 (0.13–0.17)	2,032,082
4–< 5 years	1.89 (1.83–1.95)	0.14 (0.12–0.16)	2,102,060	1.82 (1.76–1.88)	0.13 (0.11–0.15)	2,100,712	2.00 (1.94–2.06)	0.13 (0.11–0.14)	2,102,955
Total	5.86 (5.81–5.91)	0.99 (0.97–1.01)	9,822,731	5.64 (5.59–5.69)	0.94 (0.92–0.95)	9,801,189	6.17 (6.12–6.22)	1.00 (0.98–1.02)	9,831,577
RSV‐ARI									
<1 year	9.29 (9.15–9.43)	2.15 (2.08–2.22)	1,833,265	9.27 (9.13–9.41)	2.14 (2.07–2.20)	1,825,437	9.51 (9.37–9.65)	2.02 (1.96–2.09)	1,836,507
1–< 2 years	2.55 (2.48–2.62)	0.47 (0.44–0.50)	1,893,334	2.54 (2.46–2.61)	0.46 (0.43–0.49)	1,891,469	2.72 (2.65–2.80)	0.42 (0.40–0.45)	1,894,320
2–< 3 years	1.10 (1.05–1.15)	0.08 (0.07–0.10)	1,964,961	1.09 (1.05–1.14)	0.08 (0.07–0.10)	1,964,115	1.17 (1.12–1.22)	0.06 (0.05–0.07)	1,965,712
3–< 4 years	0.56 (0.53–0.59)	0.04 (0.03–0.05)	2,031,431	0.56 (0.52–0.59)	0.04 (0.03–0.05)	2,030,998	0.58 (0.55–0.62)	0.03 (0.02–0.04)	2,032,082
4–< 5 years	0.30 (0.27–0.32)	0.03 (0.02–0.04)	2,102,341	0.29 (0.27–0.32)	0.03 (0.02–0.03)	2,102,119	0.31 (0.29–0.34)	0.02 (0.01–0.02)	2,102,955
Total	2.62 (2.59–2.66)	0.52 (0.51–0.54)	9,825,333	2.61 (2.58–2.64)	0.52 (0.50–0.53)	9,814,137	2.72 (2.69–2.75)	0.48 (0.47–0.50)	9,831,577
RSV‐LRTI									
< 1 year	7.91 (7.78–8.04)	1.83 (1.77–1.89)	1,833,463	7.90 (7.78–8.03)	1.82 (1.76–1.89)	1,826,744	8.44 (8.31–8.57)	1.83 (1.77–1.90)	1,836,507
1–< 2 years	1.91 (1.85–1.98)	0.36 (0.33–0.39)	1,893,434	1.91 (1.85–1.97)	0.36 (0.33–0.39)	1,892,014	2.29 (2.23–2.36)	0.38 (0.35–0.41)	1,894,320
2–< 3 years	0.77 (0.73–0.81)	0.06 (0.05–0.07)	1,965,016	0.76 (0.73–0.80)	0.06 (0.05–0.07)	1,964,422	0.97 (0.93–1.02)	0.06 (0.05–0.07)	1,965,712
3–< 4 years	0.37 (0.34–0.40)	0.03 (0.02–0.04)	2,031,461	0.37 (0.34–0.40)	0.03 (0.02–0.04)	2,031,170	0.47 (0.44–0.50)	0.03 (0.02–0.04)	2,032,082
4–< 5 years	0.16 (0.15–0.18)	0.02 (0.01–0.02)	2,102,364	0.16 (0.14–0.18)	0.02 (0.01–0.02)	2,102,238	0.24 (0.22–0.26)	0.01 (0.01–0.02)	2,102,955
Total	2.11 (2.08–2.14)	0.43 (0.42–0.44)	9,825,739	2.10 (2.07–2.13)	0.43 (0.42–0.44)	9,816,588	2.36 (2.33–2.39)	0.44 (0.42–0.45)	9,831,577

*Note:* Reported rates are as observed in claims data and not adjusted for RSV under‐ascertainment.

*Abbreviations*: ARI, acute respiratory infection; ICU, intensive care unit; IP, inpatient; LRTI, lower respiratory tract infection; RSV, respiratory syncytial virus.

**TABLE 2:** Comparison of annual outpatient LRTI, RSV‐ARI, and RSV‐LRTI incidence estimates per 1000 person‐years by chronological age, and approach to delineate unique episodes.

	Approach 1 (30‐day washout)			Approach 2 (1 episode/year)			Approach 3 (encounter‐based)		
	Incidence (total OP) (95% CI)	Incidence (ED) (95% CI)	Risk years	Incidence (total OP) (95% CI)	Incidence (ED) (95% CI)	Risk years	Incidence (total OP) (95% CI)	Incidence (ED) (95% CI)	Risk years
LRTI									
< 1 year	250.34 (249.58–251.10)	32.93 (32.66–33.21)	1,670,025	210.15 (209.46–210.84)	28.48 (28.23–28.74)	1,683,483	368.49 (367.61–369.37)	37.48 (37.20–37.76)	1,836,507
1–< 2 years	199.63 (198.97–200.29)	24.87 (24.64–25.11)	1,744,238	168.36 (167.76–168.97)	21.74 (21.52–21.96)	1,753,617	261.46 (260.74–262.19)	27.39 (27.15–27.62)	1,894,320
2–< 3 years	148.30 (147.75–148.86)	14.42 (14.25–14.60)	1,846,276	128.06 (127.54–128.57)	12.92 (12.75–13.08)	1,855,014	176.74 (176.15–177.32)	15.28 (15.10–15.45)	1,965,712
3–< 4 years	127.73 (127.22–128.23)	10.37 (10.22–10.51)	1,924,744	111.36 (110.89–111.83)	9.36 (9.23–9.50)	1,933,155	144.28 (143.76–144.81)	10.77 (10.63–10.92)	2,032,082
4–< 5 years	110.34 (109.89–110.81)	8.06 (7.93–8.18)	2,001,526	97.04 (96.61–97.47)	7.33 (7.22–7.45)	2,010,584	126.09 (125.61–126.57)	8.39 (8.26–8.51)	2,102,955
Total	164.02 (163.75–164.28)	17.53 (17.45–17.62)	9,186,810	140.43 (140.18–140.67)	15.47 (15.39–15.55)	9,235,853	211.34 (211.05–211.63)	19.35 (19.27–19.44)	9,831,577
RSV‐ARI									
< 1 year	57.85 (57.50–58.20)	10.25 (10.11–10.40)	1,782,441	55.30 (54.95–55.64)	10.08 (9.94–10.23)	1,788,598	109.02 (108.54–109.50)	13.70 (13.53–13.87)	1,836,507
1–< 2 years	26.29 (26.05–26.52)	4.02 (3.93–4.11)	1,867,081	25.36 (25.13–25.59)	3.96 (3.87–4.05)	1,869,940	45.07 (44.77–45.38)	5.17 (5.07–5.27)	1,894,320
2–< 3 years	10.43 (10.28–10.57)	1.58 (1.53–1.64)	1,953,981	10.14 (10.00–10.29)	1.57 (1.51–1.63)	1,955,226	17.03 (16.85–17.22)	2.06 (2.00–2.13)	1,965,712
3–< 4 years	4.37 (4.28–4.46)	0.71 (0.67–0.75)	2,026,654	4.26 (4.17–4.35)	0.71 (0.67–0.74)	2,027,206	7.22 (7.10–7.34)	0.91 (0.87–0.95)	2,032,082
4–< 5 years	2.03 (1.97–2.09)	0.28 (0.26–0.30)	2,100,086	1.95 (1.89–2.01)	0.28 (0.26–0.30)	2,100,332	3.52 (3.44–3.60)	0.36 (0.34–0.39)	2,102,955
Total	19.08 (19.00–19.17)	3.17 (3.14–3.21)	9,730,243	18.36 (18.28–18.45)	3.13 (3.10–3.17)	9,741,302	34.70 (34.58–34.81)	4.23 (4.19–4.27)	9,831,577
RSV‐LRTI									
< 1 year	48.29 (47.97–48.62)	7.95 (7.82–8.08)	1,790,725	46.30 (45.98–46.61)	7.83 (7.70–7.96)	1,795,716	89.66 (89.23–90.09)	10.75 (10.60–10.90)	1,836,507
1–< 2 years	20.67 (20.46–20.87)	2.87 (2.79–2.94)	1,872,670	19.95 (19.75–20.16)	2.83 (2.76–2.91)	1,874,834	35.65 (35.39–35.92)	3.78 (3.69–3.86)	1,894,320
2–< 3 years	7.87 (7.75–8.00)	1.08 (1.03–1.12)	1,956,716	7.65 (7.53–7.78)	1.07 (1.02–1.11)	1,957,627	13.01 (12.85–13.17)	1.41 (1.36–1.46)	1,965,712
3–< 4 years	3.28 (3.20–3.36)	0.46 (0.43–0.49)	2,027,931	3.19 (3.11–3.27)	0.46 (0.43–0.49)	2,028,325	5.52 (5.42–5.62)	0.60 (0.57–0.64)	2,032,082
4–< 5 years	1.55 (1.50–1.60)	0.18 (0.16–0.20)	2,100,673	1.48 (1.43–1.54)	0.18 (0.16–0.20)	2,100,852	2.72 (2.65–2.79)	0.23 (0.21–0.25)	2,102,955
Total	15.44 (15.36–15.52)	2.36 (2.33–2.39)	9,748,715	14.87 (14.80–14.95)	2.33 (2.30–2.36)	9,757,353	27.94 (27.84–28.04)	3.19 (3.16–3.23)	9,831,577

*Note:* Reported rates are as observed in claims data and not adjusted for RSV under‐ascertainment.

*Abbreviations*: ARI, acute respiratory infection; ED, emergency department visit; LRTI, lower respiratory tract infection; OP, outpatient; RSV, respiratory syncytial virus.

RSV‐LRTI accounted for over half of all LRTI hospital admissions for children < 1 year (7.9 RSV‐LRTI versus 14.7 LRTI per 1000 person‐years), but this contribution declined for older age groups (e.g., 0.2 RSV‐LRTI versus 1.9 LRTI for age 4). Among outpatients, RSV‐LRTI accounted for about 20% of LRTI episodes among children < 1 year (48.3 versus 250.3) and had marginal contribution among older children. Overall, for every inpatient episode, we found more than seven outpatient RSV‐LRTI episodes (6–11 depending on age). Lastly, RSV‐ARI rates were typically between 10% and 20% higher than RSV‐LRTI, regardless of setting or age group, suggesting that most captured RSV‐ARI were LRTI.

### 4.1 Delineation of Unique Infection Episodes

Comparing varying approaches to delineate infection episodes, we noted marginal differences for inpatient RSV‐LRTI or RSV‐ARI between Approaches 1 and 2. Decreases in incidence rates for outpatient LRTI were slightly more pronounced when using Approach 2 versus 1, although never exceeded more than 20%. Using Approach 3 resulted in < 10% increases for inpatient incidences for any of the three outcomes versus Approach 1. For outpatient episodes, RSV‐LRTI and RSV‐ARI incidence rates almost doubled across all age groups when using Approach 3 versus 1, but this was age‐dependent for LRTI: Incidence estimates for outpatient LRTI encounters increased by 47% for infants <1 year (250.3 versus 368.5), but only 14% for 4‐year olds (110.3 versus 126.1). Findings for Approaches 2 and 3 and seasonal estimates are available in the interactive eTable 1, eTable 2, and eFigure 2 (see https://bit.ly/41bpz4u).

### 4.2 High‐Risk Stratification

**FIGURE 2:**
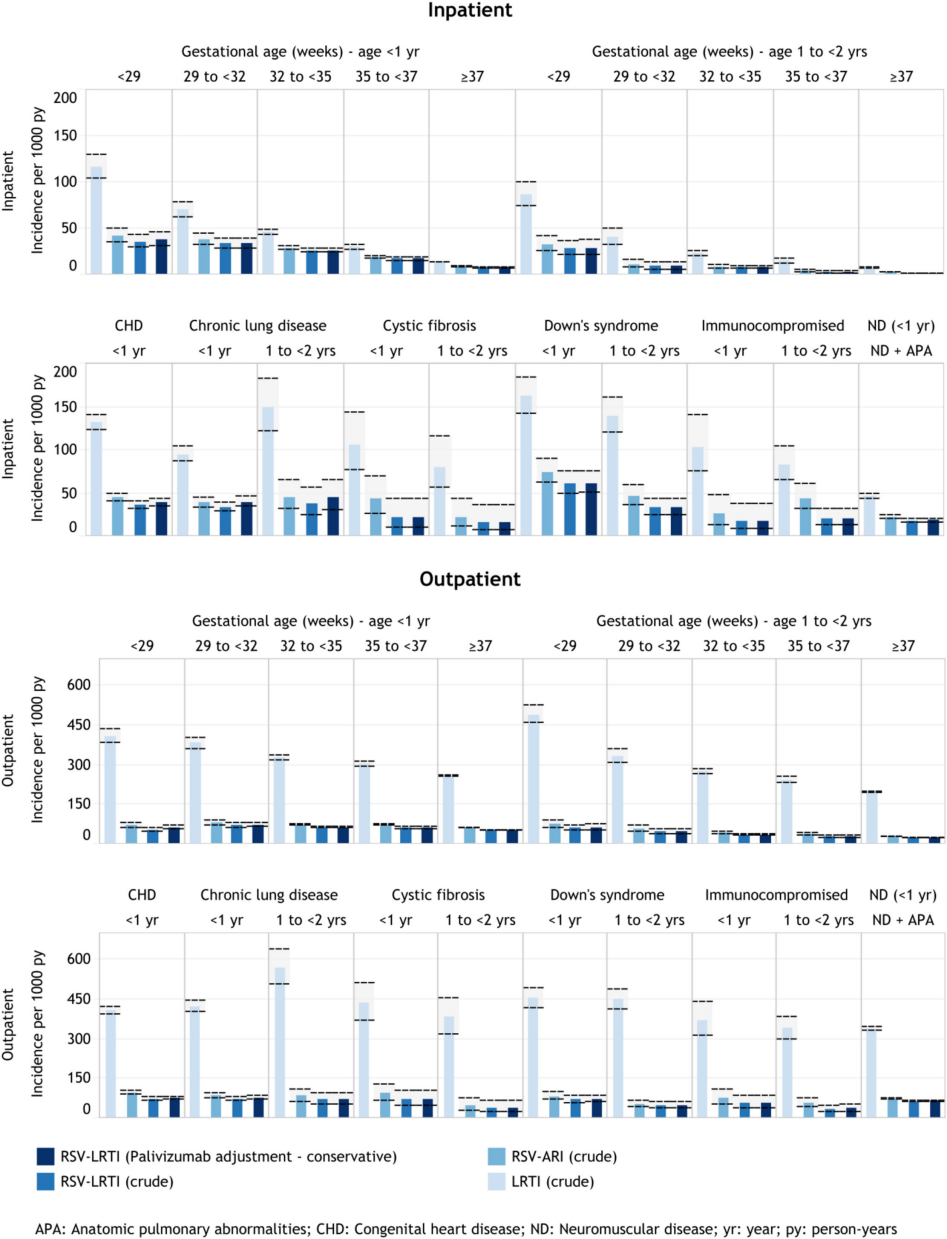
Annual crude and palivizumab‐adjusted RSV‐LRTI incidence, approach 1 (30‐day washout to delineate unique episodes) for high‐risk groups. *Note*: Reported rates are as observed in claims data and not adjusted for RSV under‐ascertainment.

As expected, RSV‐LRTI incidence rates dropped consistently with increasing gestational age with 35.6 per 1000 person‐years inpatient RSV‐LRTI episodes among infants born at < 29 weeks' gestation compared with 7.6 among term infants (Figure 2). The same GA‐specific trend was observed among children 1–< 2 years, although incidence rates of moderately premature children 1–< 2 years had approximated the risk of term infants < 1 year (e.g., 7.6 for children ≥ 1 year with gestational age 32 to < 35 versus 7.6 for term infants < 1 year). RSV‐LRTI incidence in the outpatient setting for infants was similar across gestational age groups (e.g., 54.0 for infants <29 weeks' gestation, 51.6 for term infants).

Comparing incidence rates among high‐risk medical conditions at < 1 year, children with Down syndrome had the highest RSV‐LRTI incidence (60.9 inpatient episodes per 1000 person‐years versus 7.6 for the overall population of term infants), followed by those with CHD (36.2), extreme prematurity (< 29 weeks of gestation, 27.9), and CLD (33.8). RSV‐LRTI inpatient episodes among infants with CF (21.4), immunocompromised children (17.9), or children with anatomic pulmonary abnormalities/neuromuscular disease (17.8) were more than double that of term infants (Figure 2). Interestingly, for CLD, incidence rates at 1–< 2 years were only slightly higher than at < 1 year (37.2 versus 33.7/1000 person‐years for inpatient RSV‐LRTI).

For LRTI and RSV‐ARI, trends across high‐risk conditions were largely similar to those described for RSV‐LRTI. Inpatient and outpatient incidence was up to 3% and 10% lower, respectively, for Approach 2 versus 1 across all risk groups and up to 1.8 and 3.5 times higher for Approach 3.

We noted more than a doubling when incidence rates were estimated across the core season months rather than across a full calendar year for the two RSV‐specific outcomes, and a slightly lesser increase for LRTI, across all settings and age groups. For example, the RSV‐LRTI inpatient incidence for infants in their first year of life was 66/100,000 person‐months when estimated across a year, while the rate measured over the core season months was 153 (for all contrasts between annual and seasonal rates refer to the eTables and eFigures (see https://bit.ly/41bpz4u)).

Compared with the incidence rate where the denominator represents the exact amount of each infant's time at risk during the observation period (e.g., follow‐up ended at a child's birthday for age‐specific estimates), the incidence proportions, which counted the number of children ignoring their exact at‐risk time, were about 30% lower. For example, for infants <1 year, we observed an inpatient RSV‐LRTI rate of 7.9/1000 person‐years, compared with 5.3/1000 persons with an average follow‐up time of 8.0 months). (interactive eTable3 [see https://bit.ly/41bpz4u]).

### 4.3 Immunoprophylaxis‐Adjusted Incidence Rates

**FIGURE 3:**
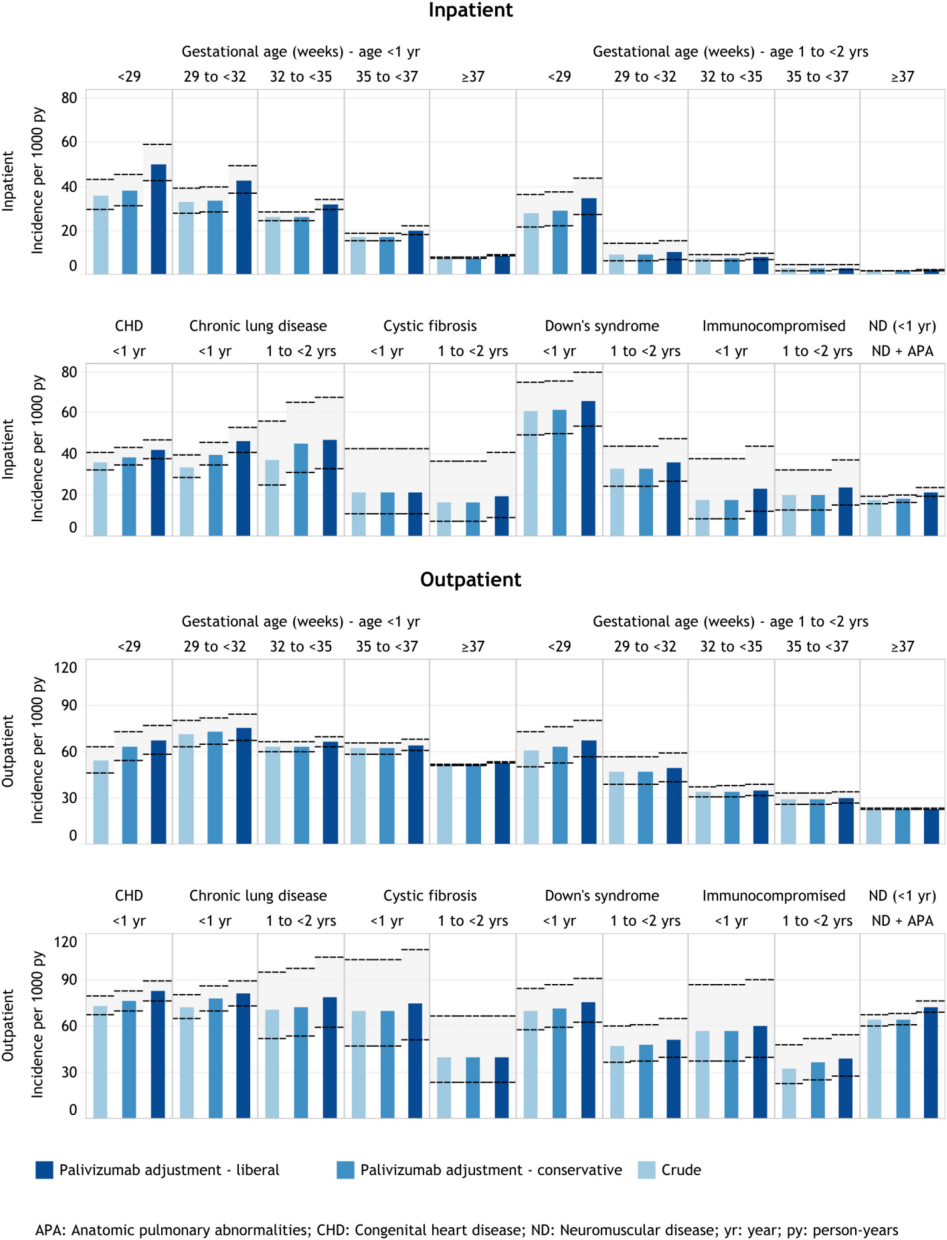
Comparison of conservative and liberal palivizumab adjustment for annual crude and palivizumab‐adjusted RSV‐LRTI incidence, approach 1 (30‐day washout to delineate unique episodes) for high‐risk groups. *Note*: Reported rates are as observed in claims data and not adjusted for RSV under‐ascertainment.

Adjustments for palivizumab exposure had marginal effects (i.e., typically < 10%) on incidence estimates except for children with CLD where RSV‐LRTI inpatient incidence estimates increased from 33.7 to 39.7/1000 person‐years for infants < 1 year and from 37.2 to 44.9 for those 1–< 2 years (Figure 2). The assumption that the first 30 days after hospital discharge were exposed to palivizumab resulted in consistently increased incidence estimates (e.g., for extreme preterm infants, 35.6 in the crude analysis, 37.9 using our adjustment in the main analysis, and 50.0 using the adjustment in the sensitivity analysis) (see Figure 3 and interactive eTable2 [see https://bit.ly/41bpz4u]).

Our NREVSS‐based RSV‐LRTI prediction model estimated that 42.2% of all true RSV‐LRTI episodes were diagnosed and coded, reflecting undercoding of RSV‐LRTI episodes among children enrolled under commercial insurance [26]. Using the pooled incidence rate across all settings and all children, (17.5/1000 person‐years, 2.1 for inpatient and 15.4 for outpatient), adjustment for this false negative rate resulted in an adjusted incidence rate of 41.5/1000 person‐years.

## 5 Discussion

In this study, we estimated RSV‐LRTI incidence rates among children in inpatient and outpatient settings. For context, we compared RSV‐LRTI incidences with LRTI and RSV‐ARI incidences and evaluated the impact of three methodological approaches to delineate unique infection episodes. Several findings are noteworthy. First, regardless of setting and measurement approach, RSV‐LRTI incidence rates dropped steadily with increasing age, which is consistent with previous reports [27, 28, 29]. Age‐related decreases were less pronounced for LRTI than for RSV‐specific outcomes, which confirms previously described age‐related changes in pathogens [29, 30, 31]. RSV‐LRTI accounted for more than half of all LRTI hospital admissions and one‐fifth of all outpatient episodes for infants, but this contribution dropped appreciably for older ages. Of note, the presence of RSV‐LRTI in the outpatient setting was high, with rates consistently more than five times that of inpatient rates.

Second, when expanding our definition of RSV‐associated disease to lower and upper respiratory tract infections (RTIs), we noted increases in incidence by about 10%–20%, regardless of setting and age group. While this might be surprising, it is likely a reflection of the proportionally smaller contribution of RSV to upper RTIs when compared with LRTI [32, 33], combined with lower testing rates for RSV.

Third, RSV‐LRTI inpatient incidence among infants dropped with increasing gestational age. While rates continued to decline in the second year of life, the incidence among moderately premature children had by then approximated the risk of term infants < 1 year, which is consistent with previous reports [34]. Interestingly, RSV‐LRTI rates in the outpatient setting for infants were similar across gestational age strata but varied several fold in the inpatient setting, likely because a larger proportion of severe episodes requiring hospitalization occur among preterm infants [35]. Infants with Down syndrome had the highest RSV‐LRTI risk with incidence rates eight times higher than for term infants, followed by those with CHD, extreme prematurity, and CLD, which is consistent with previous literature [15]. RSV‐LRTI inpatient rates among infants with the remaining high‐risk conditions were smaller but still double that of healthy term infants. The counter‐intuitive observation regarding higher inpatient RSV‐LRTI for infants with CLD at age ≥ 1 year when compared with < 1 year is likely due to our measurement following the AAP recommendation, which requires, besides the diagnosis active treatment in the second year but not the first, potentially selecting children with more severe disease [20].

While many studies have reported RSV rates for specific high‐risk groups, we only found one that allowed comparisons across the various conditions [36]. Based on an analysis of the US National Inpatient Sample, differences in RSV‐LRTI hospitalization rates were similar with the exception of CHD, which was associated with higher incidence rates than CLD. However, definitions relied on ICD coding on hospital encounters only and could not ascertain whether the condition required treatment, as specified in the AAP criteria.

Fourth, for RSV specific outcomes, use of a 30‐day washout period versus restriction to only one episode per season had only marginal impact on incidence rates. While this confirms the low contribution of recurrent episodes to overall disease burden, we described previously that the risk for reinfection among children with a first infection does not appear to drop [37]. In the outpatient setting, counts of every medical encounter doubled incidence rates when compared with delineation with a 30‐day washout period. Comparisons of these approaches suggest that wash‐out periods larger than 30 days are not necessary to avoid double‐counting infections.

Adjustments for palivizumab exposure had mostly marginal effects except for children with CLD and when assuming the first 30 days after hospital discharge were exposed to palivizumab, which is a function of relatively small exposure periods compared with the total follow‐up time and only moderate protection.

In contrast to most previous reports, we estimated annual and seasonal incidence rates using an open cohort design that allowed infants to enter and exit a specific stratum as they met relevant criteria. This approach also allowed direct comparison of annual and seasonal rates. The risk for RSV‐LRTI or RSV‐ARI during the core season is double that of annual risk estimates indicating that RSV infections cluster during the RSV season. This has implications for intervention design and public health surveillance. While interventions to reduce RSV infection rate may be best conducted during core seasons to maximize impact, annual rates would better represent the epidemiologic burden of RSV over an entire year.

Other studies have been agnostic of the actual follow‐up time contributed by individuals and reported incidence proportions [38]. To allow comparisons, we replicated this approach and also measured the average follow‐up time each person contributed over a year. Depending on the risk stratum, children contributed between 6 and 8 months of follow‐up during a study year. This resulted in appreciable underestimates of disease risk when reported as incidence proportions compared with incidence rates, which consider the actual time they contributed to the observation window.

This study reports in detail on the epidemiology of medically attended RSV infections, across various settings and risk groups, which were explicitly defined based on AAP recommendations. Incidence estimates were aggregated across multiple study years to derive stable estimates that can inform risk–benefit as well as cost–benefit assessments and further guide recommendations for immunoprophylaxis as new agents are introduced to the market.

In measuring RSV‐specific outcomes, we relied on presence of pathogen coding on the clinical encounter claim, which not only requires a clinician's decision to test for RSV but also integration of the positive test result in the insurance claim. This results in a high measure of specificity, but lower sensitivity, which further varies by setting, disease severity and patient characteristics (e.g., age) [39]. To overcome this issue, previous studies have used the seasonal variation of RSV to model its contribution to LRTI episodes and then adjusted for RSV‐underestimates in claims data [40, 41]. Because NRVESS data does not distinguish test results by setting or patient characteristics, we conducted our adjustment only across the entire study population, assuming that this more closely reflects the population that is represented in NREVSS and thus, produces valid inferences based on the modeling results. Based on this, we found that 42% of predicted RSV‐LRTI were coded in claims data.

Finally, we note that our study was confined to privately insured children and cannot make inferences about the disease burden among those in public insurance.

## 6 Conclusion

This study provides population‐based estimates of medically attended RSV infections across age groups and high‐risk strata and tested the impact of several methodological approaches. We found a substantial contribution of RSV to overall LRTI among infants and a decreasing role as age increases. High‐risk groups had incidence rates at least double that of healthy children. Across all age groups, most RSV cases were LRTIs and outpatient RSV‐LRTI incidence was more than five times higher than inpatient rates. Inpatient incidence rates were similar with and without imposed wash‐out periods to define unique episodes. Measurement of incidence across the core season yielded rates twice as high as annual rates. Modeling against test positivity rates suggested that about 42% of episodes are captured in claims data. Results allow granular assessments of disease burden to guide recommendations for new RSV prevention strategies.

## Author Contributions

Two MSD employees were co‐authors and contributed to the study design and writing the manuscript.

## Conflicts of Interest

Almut Winterstein has received research funding from the NIH, AHRQ, PCORI, FDA, and the state of Florida and received honoraria as a consultant from Arbor Pharmaceuticals, Ipsen, and Genentech Inc, none of which is related to this work. Yoonyoung Choi and Lyn Finelli are employees at Merck Sharp & Dohme LLC, a subsidiary of Merck & Co., Inc., Rahway, NJ, USA. Sabina Nduaguba, Phuong Tran, Renata Shih, and Yanning Wang have no conflicts of interest to declare.

### Peer Review

The peer review history for this article is available at https://www.webofscience.com/api/gateway/wos/peer‐review/10.1111/irv.70094.

## Supporting information


**Data S1** Supplementary Information.

**Data S2** Supplementary Information.

## Data Availability

The data that support the findings of this study are not available from the authors because restrictions apply to the availability of these data, which were provided by Merative (https://www.merative.com/documents/merative‐marketscan‐research‐databases), and so are not publicly available.

## References

[1] Y. Li , X. Wang , D. M. Blau , et al., “Global, Regional, and National Disease Burden Estimates of Acute Lower Respiratory Infections Due to Respiratory Syncytial Virus in Children Younger Than 5 Years in 2019: A Systematic Analysis,” Lancet (London, England) 399, no. 10340 (2022): 2047–2064, 10.1016/s0140-6736(22)004780. 35598608 PMC7613574

[2] S. Joffe , G. T. Ray , G. J. Escobar , S. B. Black , and T. A. Lieu , “Cost‐Effectiveness of Respiratory Syncytial Virus Prophylaxis Among Preterm Infants,” Pediatrics 104, no. 3 Pt 1 (1999): 419–427. 10469764 10.1542/peds.104.3.419

[3] T. P. Stevens , R. A. Sinkin , C. B. Hall , W. M. Maniscalco , and K. M. McConnochie , “Respiratory Syncytial Virus and Premature Infants Born at 32 Weeks' Gestation or Earlier: Hospitalization and Economic Implications of Prophylaxis,” Archives of Pediatrics & Adolescent Medicine 154, no. 1 (2000): 55–61. 10632251

[4] N. O. Elhassan , M. E. Sorbero , C. B. Hall , T. P. Stevens , and A. W. Dick , “Cost‐Effectiveness Analysis of Palivizumab in Premature Infants Without Chronic Lung Disease,” Archives of Pediatrics & Adolescent Medicine 160, no. 10 (2006): 1070–1076, 10.1001/archpedi.160.10.1070. 17018467

[5] W. A. Prescott, Jr. , F. Doloresco , J. Brown , and J. A. Paladino , “Cost Effectiveness of Respiratory Syncytial Virus Prophylaxis: A Critical and Systematic Review,” PharmacoEconomics 28, no. 4 (2010): 279–293, 10.2165/11531860-000000000-00000. 20131925

[6] C. A. Reeve , J. S. Whitehall , P. G. Buettner , R. Norton , D. M. Reeve , and F. Francis , “Cost‐Effectiveness of Respiratory Syncytial Virus Prophylaxis With Palivizumab,” Journal of Paediatrics and Child Health 42, no. 5 (2006): 253–258, 10.1111/j.14401754.2006.00850.x. 16712554

[7] T. I. Shireman and K. S. Braman , “Impact and Cost‐Effectiveness of Respiratory Syncytial Virus Prophylaxis for Kansas Medicaid's High‐Risk Children,” Archives of Pediatrics & Adolescent Medicine 156, no. 12 (2002): 1251–1255. 12444839 10.1001/archpedi.156.12.1251

[8] S. Wegner , J. J. Vann , G. Liu , et al., “Direct Cost Analyses of Palivizumab Treatment in a Cohort of at‐Risk Children: Evidence From the North Carolina Medicaid Program,” Pediatrics 114, no. 6 (2004): 1612–1619, 10.1542/peds.20040959. 15574623

[9] L. B. Weiner , A. S. Masaquel , M. J. Polak , and P. J. Mahadevia , “Cost‐Effectiveness Analysis of Palivizumab Among Pre‐Term Infant Populations Covered by Medicaid in the United States,” Journal of Medical Economics 15, no. 5 (2012): 997–1018, 10.3111/13696998.2012.672942. 22435648

[10] L. E. Yount and W. T. Mahle , “Economic Analysis of Palivizumab in Infants With Congenital Heart Disease,” Pediatrics 114, no. 6 (2004): 1606–1611, 10.1542/peds.20040224. 15574622

[11] C. Hampp , T. Kauf , A. Saidi , and A. Winterstein , “Cost‐Effectiveness of Respiratory Syncytial Virus Prophylaxis in Various Indications,” Archives of Pediatrics & Adolescent Medicine 165, no. 6 (2011): 498–505, 10.1001/archpediatrics.2010.298. 21300647

[12] American Academy of Pediatrics Committee on Infectious Diseases, American Academy of Pediatrics Bronchiolitis Guidelines Committee , “Updated Guidance for Palivizumab Prophylaxis Among Infants and Young Children at Increased Risk of Hospitalization for Respiratory Syncytial Virus Infection,” Pediatrics 134, no. 2 (2014): 415–420, 10.1542/peds.20141665. 25070315

[13] Committee on Infectious Diseases , “From the American Academy of Pediatrics: Policy Statements—Modified Recommendations for Use of Palivizumab for Prevention of Respiratory Syncytial Virus Infections,” Pediatrics 124, no. 6 (2009): 1694–1701, 10.1542/peds.20092345. 19736258

[14] P. Manzoni , J. Figueras‐Aloy , E. A. F. Simões , et al., “Defining the Incidence and Associated Morbidity and Mortality of Severe Respiratory Syncytial Virus Infection Among Children With Chronic Diseases,” Infectious Disease and Therapy 6, no. 3 (2017): 383–411, 10.1007/s40121-017-0160-3. PMC5595774 28653300

[15] A. A. Beckhaus and J. A. Castro‐Rodriguez , “Down Syndrome and the Risk of Severe RSV Infection: A Meta‐Analysis,” Pediatrics 142, no. 3 (2018): e20180225, 10.1542/peds.20180225. 30093540

[16] AAP Publications Reaffirmed,” Pediatrics 144 (2019): e2023062145, 10.1542/peds.20191767. 31358666

[17] Centene Corporation . Synagis® (Palivizumab) 2021–2022 Authorization Guideline. Centene Corporation. June 14, 2023. accessed June 14, 2023, https://www.coordinatedcarehealth.com/content/dam/centene/Coordinated%20Care/provider/PDFs/Pharmacy/508‐Synagis‐Auth‐Guidelines.pdf.

[18] A. G. Winterstein , E. Eworuke , D. Xu , and P. Schuler , “Palivizumab Immunoprophylaxis Effectiveness in Children With Cystic Fibrosis,” Pediatric Pulmonology 48 (2012): 874–884, 10.1002/ppul.22711. 23139089 PMC7167886

[19] A. G. Winterstein , C. Hampp , and A. Saidi , “Effectiveness of Palivizumab Prophylaxis in Infants and Children in Florida,” Pharmacoepidemiology and Drug Safety 21, no. 1 (2012): 53–60, 10.1002/pds.2246. 21919115

[20] Y. Choi , H. C. Meissner , C. Hampp , H. Park , B. Brumback , and A. G. Winterstein , “Calibration of Chronic Lung Disease Severity as a Risk Factor for Respiratory Syncytial Virus Hospitalization,” Journal of the Pediatric Infectious Diseases Society 10, no. 3 (2021): 317–325, 10.1093/jpids/piaa107. 32978942

[21] A. Sarayani , X. Wang , T. N. Thai , Y. Albogami , N. Jeon , and A. G. Winterstein , “Impact of the Transition From ICD‐9‐CM to ICD‐10‐CM on the Identification of Pregnancy Episodes in US Health Insurance Claims Data,” Clinical Epidemiology 12 (2020): 1129–1138, 10.2147/clep.S269400. 33116906 PMC7571578

[22] K. J. Rothman , S. Greenland , and T. L. Lash . Modern Epidemiology. 3rd edition, thoroughly revised and updated. ed. Wolters Kluwer Health/Lippincott Williams & Wilkins; 2008.

[23] Palivizumab, a Humanized Respiratory Syncytial Virus Monoclonal Antibody, Reduces Hospitalization From Respiratory Syncytial Virus Infection in High‐Risk Infants. The IMpact‐RSV Study Group,” Pediatrics 102, no. 3 Pt 1 (1998): 531–537. 9738173

[24] T. F. Feltes , A. K. Cabalka , H. C. Meissner , et al., “Palivizumab Prophylaxis Reduces Hospitalization due to Respiratory Syncytial Virus in Young Children With Hemodynamically Significant Congenital Heart Disease,” Journal of Pediatrics 143, no. 4 (2003): 532–540, 10.1067/s0022-3476(03)004542. 14571236

[25] T. Andabaka , J. W. Nickerson , M. X. Rojas‐Reyes , J. D. Rueda , V. Bacic Vrca , and B. Barsic , “Monoclonal Antibody for Reducing the Risk of Respiratory Syncytial Virus Infection in Children,” Cochrane Database of Systematic Reviews , no. 4 (2013): Cd006602, 10.1002/14651858.CD006602.pub4. 23633336

[26] S. Nduaguba , P. Tran , and A. Winterstein . Correction of Diagnostic Coding‐Based RSV Incidence Using NREVSS Data. 2022:266. 10.1186/s12879-024-09474-y PMC11191139 38907351

[27] C. B. Hall , G. A. Weinberg , M. K. Iwane , et al., “The Burden of Respiratory Syncytial Virus Infection in Young Children,” New England Journal of Medicine 360, no. 6 (2009): 588–598, 10.1056/NEJMoa0804877. 19196675 PMC4829966

[28] E. Goldstein , L. Finelli , A. O'Halloran , et al., “Hospitalizations Associated With Respiratory Syncytial Virus and Influenza in Children, Including Children Diagnosed With Asthma,” Epidemiology (Cambridge, Mass.) 30, no. 6 (2019): 918–926, 10.1097/ede.0000000000001092. 31469696 PMC6768705

[29] F. T. Bourgeois , C. Valim , A. J. McAdam , and K. D. Mandl , “Relative Impact of Influenza and Respiratory Syncytial Virus in Young Children,” Pediatrics 124, no. 6 (2009): e1072–e1080, 10.1542/peds.20083074. 19933730 PMC3374864

[30] S. Jain , D. J. Williams , S. R. Arnold , et al., “Community‐Acquired Pneumonia Requiring Hospitalization Among U.S. Children,” New England Journal of Medicine 372, no. 9 (2015): 835–845, 10.1056/NEJMoa1405870. 25714161 PMC4697461

[31] B. Rha , A. T. Curns , J. Y. Lively , et al., “Respiratory Syncytial Virus‐Associated Hospitalizations Among Young Children: 2015–2016,” Pediatrics 146, no. 1 (2020): e20193611, 10.1542/peds.20193611. 32546583 PMC12874392

[32] J. A. Patel , D. T. Nguyen , K. Revai , and T. Chonmaitree , “Role of Respiratory Syncytial Virus in Acute Otitis Media: Implications for Vaccine Development,” Vaccine 25, no. 9 (2007): 1683–1689, 10.1016/j.vaccine.2006.10.045. 17156899 PMC1828634

[33] T. Chonmaitree , K. Revai , J. J. Grady , et al., “Viral Upper Respiratory Tract Infection and Otitis Media Complication in Young Children,” Clinical Infectious Diseases : An Official Publication of the Infectious Diseases Society of America 46, no. 6 (2008): 815–823, 10.1086/528685. 18279042 PMC2744371

[34] A. G. Winterstein , C. A. Knox , P. Kubilis , and C. Hampp , “Appropriateness of Age Thresholds for Respiratory Syncytial Virus Immunoprophylaxis in Moderate‐Preterm Infants: A Cohort Study,” JAMA Pediatrics 167, no. 12 (2013): 1118–1124, 10.1001/jamapediatrics.2013.2636. 24126903

[35] E. R. Packnett , I. H. Winer , A. Oladapo , and M. Wojdyla , “Risk of RSV‐Related Hospitalization Is Associated With Gestational age in Preterm (Born at 29–34 wGA) Infants Without Outpatient Palivizumab Administration,” Human Vaccines & Immunotherapeutics 19, no. 2 (2023): 2252289, 10.1080/21645515.2023.2252289. 37828711 PMC10578184

[36] A. Doucette , X. Jiang , J. Fryzek , J. Coalson , K. McLaurin , and C. S. Ambrose , “Trends in Respiratory Syncytial Virus and Bronchiolitis Hospitalization Rates in High‐Risk Infants in a United States Nationally Representative Database, 1997–2012,” PLoS ONE 11, no. 4 (2016): e0152208, 10.1371/journal.pone.0152208. 27050095 PMC4822775

[37] S. O Nduaguba , P. T. Tran , Y. Choi , and A. G. Winterstein . “Respiratory Syncytial Virus Reinfections Among Infants and Young Children in the United States, 2011–2019,” PLoS One 18, no. 2 (2023): e0281555, 10.1371/journal.pone.0281555. 36795639 PMC9934310

[38] M. Suh , N. Movva , X. Jiang , et al., “Respiratory Syncytial Virus Burden and Healthcare Utilization in United States Infants <1 Year of Age: Study of Nationally Representative Databases, 2011–2019,” Journal of Infectious Diseases 226, no. Suppl 2 (2022): S184–s194, 10.1093/infdis/jiac155. 35968879 PMC9377028

[39] P. T. Tran , S. O. Nduaguba , V. Diaby , Y. Choi , and A. G. Winterstein , “RSV Testing Practice and Positivity by Patient Demographics in the United States: Integrated Analyses of MarketScan and NREVSS Databases,” BMC Infectious Diseases 22, no. 1 (2022): 681, 10.1186/s12879-022-07659-x. 35941563 PMC9360654

[40] G. Matias , R. Taylor , F. Haguinet , C. Schuck‐Paim , R. Lustig , and V. Shinde , “Estimates of Hospitalization Attributable to Influenza and RSV in the US During 1997–2009, by Age and Risk Status,” BMC Public Health 17, no. 1 (2017): 271, 10.1186/s12889-017-4177-z. 28320361 PMC5359836

[41] H. Zhou , W. W. Thompson , C. G. Viboud , et al., “Hospitalizations Associated With Influenza and Respiratory Syncytial Virus in the United States, 1993–2008,” Clinical Infectious Diseases : An Official Publication of the Infectious Diseases Society of America 54, no. 10 (2012): 1427–1436, 10.1093/cid/cis211. 22495079 PMC3334364

