# Supplementary material for: Respiratory Syncytial Virus Incidence in Young Children in the United States: Impact of Methodologies and Patient Characteristics
Source: Influenza Other Respir Viruses. 2025 Apr 3;19(4):e70094. doi: 10.1111/irv.70094 (PMC11966010; doi:10.1111/irv.70094)
Supplement: Supplementary file 1 — Data S1 Supplementary Information. [file IRV-19-e70094-s002.docx]

**Supplemental Materials**

**Supplemental Methods**

**Definition of high-risk groups**

*General rules:* High-risk clinical conditions were determined any time before or during a risk period unless the definition requires updates on status (e.g., receiving chemotherapy). We required children to have at least 1 inpatient or 2 outpatient encounters 30 days apart. The earliest encounter was considered the index date to assign children to a high-risk condition.

**Gestational Age** – We applied an algorithm to define gestational age (GA) using ICD-10-CM medical encounter claims. First, we used ICD-10-CM codes for GA on medical encounters of infants (age=0). Among infants without any encounter indicating GA, we linked them with their mothers to obtain GA information using our validated pregnancy algorithm.^23^ In short, we identified unique deliveries for live births based on medical encounters of women of child-bearing age. To be linked, infants had to have the same family ID, their first medical encounter claim, or health plan enrollment date had to be within 30 days before and 30 days after of the mother’s delivery claim, and infants’ age was zero at the time of the delivery claim. If infants had conflicting GA information, we retained the smaller GA. The remaining infants, who did not have their own claims indicating GA or who were not linked to their mothers were assigned a GA ≥ 37 weeks.

**Congenital heart disease** – Congenital heart disease (CHD) diagnoses were categorized into cyanotic and acyanotic CHD. If patients had encounters with IDC codes indicating cyanotic CHD and later had acyanotic codes, all periods were considered cyanotic CHD. If a patient had acyanotic diagnoses first and cyanotic diagnoses later, the patient was considered to be acyanotic CHD until they had the first diagnosis for cyanotic CHD and the period thereafter was considered cyanotic. Acyanotic patients were required to have active medication use (days of supply >0) for CHD to be included. Exposure time was calculated from dispensing day plus days of supply (**Supplemental Excel File 1, eTable 5)**.

**Chronic lung disease** – Patients were included if they had a GA of <32 weeks at birth, were <2 years old, and had at least 1 inpatient diagnosis for chronic lung disease (CLD) or 2 related outpatient diagnoses 30 days apart. Further, they were required to have at least one inpatient or outpatient diagnostic claim for oxygen dependence or respiratory distress syndrome or procedure code for supplemental oxygen within 90 days of birth. This formed the core cohort from which separate cohorts were created based on age and period. The annual 0-year age cohort was required to have oxygen exposure within the first 28 days of birth. The annual 1-year age cohort was created from the core cohort and was further required to have diuretic, oxygen exposure, or more than 45 cumulative days’ supply of steroid medication in a 90-day window. Diuretic, oxygen, and steroid exposure were assessed within 180 days before entering the age cohort. Steroid prescription fills were assessed as a time-varying exposure (**Supplemental Excel File 1, eTable 6 and eTable 7**).

**Anatomic Pulmonary Abnormalities/Neuromuscular Disease** – Patients with these two sets of conditions were grouped into one cohort due to their low prevalence and the commonalities in affected organs. Patients were included if they were less than 1 year and had an inpatient diagnosis or 2 outpatient diagnoses 30 days apart (**Supplemental Excel File 1, eTable 9 and eTable 10**).

**Immunocompromise** – immunocompromised children were diagnosed with severe combined immunodeficiency (SCID), acquired immune deficiency syndrome (AIDS), had received a transplant (solid organ transplant or hematopoietic stem cell transplantation), or had another immunodeficiency disorders.^1^ Patients were included if they were less than 2 years and had an inpatient diagnosis or 2 outpatient diagnoses 30 days apart (**Supplemental Excel File 1, eTable 8**).

**Chemotherapy** – Patients with at least one medical encounter for chemotherapy administration or a prescription fill for at least one chemotherapy medication were included. Medical encounters with chemotherapy administration were assigned an exposure duration of 14 days while the days’ supply recorded in the prescription file were retained for dispensed chemotherapy. Start and end dates for each chemotherapy cycle were, respectively, defined as the earliest and latest day covered by any chemotherapy medication with no gap allowed. A new cycle begins on the date coverage is re-initiated following a gap. Chemotherapy medications were identified from medications classified as antineoplastic agents (antineoplastics) in Redbook supplemented by the AHFS^2,342^ and SEER^43^ lists. Included patients were required to be less than 2 years old and be exposed to at least one chemotherapy medication. Only durations of active chemotherapy exposure were included as risk periods (**Supplemental Excel File 1, eTable 11)**.

**Cystic Fibrosis** and **Down syndrome:**

Children <2 years of age with at least 1 inpatient or 2 outpatient diagnosis codes (30 days apart) of cystic fibrosis or Down syndrome were included. Because the conditions are chronic, children were considered to have cystic fibrosis or Down syndrome from the first diagnosis toward the entire follow-up (**Supplemental Excel File 1, eTable 5)**.

## Supplemental files

**Excel File 1.** Codes and medications for operationalization definitions of study conditions

**Tableau (see** [**https://bit.ly/41bpz4u**](https://bit.ly/41bpz4u)**)**

**eTable 1: Crude Incidence Rates**

Description: LRTI, RSV-ARI, and RSV-LRTI incidence by different patient strata and approaches across clinical settings and annual/seasonal RSV season. Unit is risk-month to facilitate comparison between annual and core season estimates.

**eTable 2: Adjusted Incidence Rates**

Description: RSV-LRTI incidence with palivizumab adjustment by conservative and liberal approaches for high-risk groups by approach across clinical settings and annual/seasonal RSV season. Unit is risk-month to facilitate comparison between annual and core season estimates.

**eTable 3: Crude Incidence Proportions**

Description: LRTI, RSV-ARI, and RSV-LRTI incidence rate and proportion by different patient strata.

**eFigure1: Crude Incidence Rates**

Description: Comparison of LRTI, RSV-ARI, and RSV-LRTI incidence estimate by chronological age and 3 approaches across clinical settings and annual/seasonal RSV season

**eFigure2. Crude & Adjusted Incidence Rates**

Description: Extension of Figure 2 – Annual/seasonal crude and palivizumab-adjusted RSV-LRTI incidence by 3 approaches for high-risk groups.

**REFERENCES**

1. American Academy of Allergy and Asthma & Immunology. Immunodeficiencies Disease Codes. June 14, 2023. <https://www.aaaai.org/Aaaai/media/MediaLibrary/PDF%20Documents/Practice%20Management/finances-coding/ICD-10-Codes-Immunodeficiencies.pdf>

2. American Society of Health-System Pharmacists. AHFS Pharmacologic-Therapeutic Classification System. August 16, 2022. Accessed August 16, 2022. <https://www.ashp.org/products-and-services/database-licensing-and-integration/ahfs-therapeutic-classification>

3. National Cancer Institute. SEER*Rx Interactive Antineoplastic Drugs Database. August 16, 2022. Accessed August 16, 2022. <https://seer.cancer.gov/seertools/seerrx/>
